# Eye movement desensitisation and reprocessing for post-traumatic stress in survivors of critical illness (EMERALD): a mixed-methods, randomised, single-blind, parallel group-controlled, feasibility trial

**DOI:** 10.1016/j.eclinm.2026.104082

**Published:** 2026-07-13

**Authors:** Andrew Bates, Rebecca Cusack, Hannah Golding, Helen Moyses, Hazel Southam, Sophie Rushbrook, Julie Highfield, Natalie Pattison, David S. Baldwin, Michael P.W. Grocott

**Affiliations:** a NIHR Southampton Biomedical Research Centre, University Hospital Southampton NHS Foundation Trust, Southampton, UK; b Clinical and Experimental Sciences, Faculty of Medicine, University of Southampton, Southampton, UK; c General Intensive Care Unit, University Hospital Southampton NHS Foundation Trust, Southampton, United Kingdom; d Independent Patient and Public Involvement (PPI) Contributor, Southampton, UK; e Intensive Psychological Therapies Service, Dorset Healthcare University NHS Foundation Trust, Poole, Dorset, UK; f Clinical Health and Staff Psychology, Gloucestershire Hospitals NHS Foundation Trust, Gloucester, UK; g University of Hertfordshire, East and North Hertfordshire NHS Trust, Hatfield, UK; h Mood Disorders Service, Hampshire and Isle of Wight Healthcare NHS Foundation Trust, Southampton, UK; i Department of Psychiatry and Mental Health, University of Cape Town, Cape Town, South Africa

**Keywords:** Stress disorders, Post-traumatic, Critical illness, Intensive care units, Survivors, Eye movement desensitization reprocessing, Feasibility studies

## Abstract

**Background:**

Eye movement desensitisation and reprocessing (EMDR) is a guideline-recommended treatment for post-traumatic stress disorder (PTSD), but evidence in survivors of critical illness remains limited. We assessed the feasibility, acceptability, and safety of EMDR for critical care survivors with clinically significant post-traumatic stress symptoms, and generated exploratory clinical outcome estimates to inform a future definitive trial.

**Methods:**

We conducted a mixed-methods, randomised, single-blind, parallel group-controlled, feasibility trial at three UK National Health Service hospitals. Adults (≥18 years) with an intensive care stay >24 h were approached before hospital discharge and followed within an observational cohort. At 2–3 months, participants were screened for post-traumatic stress symptoms (Impact of Event Scale–Revised); those scoring ≥22 were invited and randomly assigned (1:1) to EMDR plus treatment as usual (TAU) or TAU. EMDR comprised up to 16 sessions delivered face-to-face or online by accredited therapists. Primary outcomes were feasibility (recruitment, retention, intervention uptake, fidelity) and safety. Symptoms were assessed using Clinician-administered PTSD Scale for Diagnostic and Statistical Manual-5 (CAPS-5) at 3 and 12 months. Analyses followed intention-to-treat principles. The trial was registered on ClinicalTrials.gov (NCT05591625) and is closed to recruitment.

**Findings:**

Between Feb 20, 2023, and May 13, 2024, 160 patients were recruited to the observational cohort; 40 were randomised, with 20 allocated to EMDR plus TAU and 20 to TAU. Median age was 59.5 years (IQR 52.0–66.0); 21 participants (53%) were female and 19 (48%) were male. At 12 months, 39 (98%, 95% CI 86.8–99.9) of 40 randomised participants completed CAPS-5 follow-up. In the EMDR plus TAU group, 18 (90%, 95% CI 68.3–98.8) of 20 participants initiated treatment; mean sessions attended was 10.4 (SD 7.0), and 15 (75%) of 20 completed a full therapeutic course. Mean CAPS-5 score change was −15.6 (SD 12.5) in the EMDR plus TAU group and −1.6 (SD 11.8) in the TAU group, giving an exploratory unadjusted between-group difference of −14.1 points (95% CI −22.0 to −6.2). No treatment-related serious adverse events were identified.

**Interpretation:**

A staged trial pathway of symptom screening, clinician-rated PTSD assessment, randomisation, and EMDR delivery was feasible and broadly acceptable in survivors of critical illness with clinically significant post-traumatic stress symptoms. Exploratory clinician-rated PTSD outcome estimates were hypothesis-generating and support progression to a definitive multicentre trial, but should not be interpreted as evidence of treatment effectiveness.

**Funding:**

Andrew Bates was funded by National Institute for Health and Care Research Clinical Doctoral Research fellowship (grant number: NIHR302160).

**Research in context**

**Evidence before this study**

We searched MEDLINE, Embase, PsycINFO, CINAHL, and Web of Science for reviews and studies published from database inception to Dec 18, 2025, without language restrictions. Search terms combined concepts relating to intensive care, critical illness survivorship, psychological interventions, post-traumatic stress disorder (PTSD), anxiety, depression, and quality of life. Existing reviews, including a Cochrane review of critical illness follow-up and recovery interventions, describe a heterogeneous literature dominated by multicomponent recovery programmes, follow-up clinics, patient diaries, rehabilitation interventions, and low-intensity psychological approaches. Effects on PTSD and related mental health outcomes are inconsistent or uncertain, and studies are commonly limited by small sample sizes, heterogeneous populations, variable timing of intervention delivery, reliance on self-reported symptom measures, and short or inconsistent follow-up. A recent multicentre randomised trial of a brief general practitioner-led narrative exposure intervention reported modest reductions in PTSD symptoms after critical care, suggesting growing evidence for trauma-informed approaches while leaving uncertainty about specialist trauma-focused therapies, staged screening pathways, and clinician-rated PTSD outcomes. Few trials have targeted critical care survivors with clinically significant post-traumatic stress symptoms, and few have evaluated trauma-focused psychological therapy within a randomised design supported by clinician-rated PTSD assessment.

**Added value of this study**

EMERALD evaluated the feasibility and acceptability of a staged trauma-focused trial pathway for critical illness survivors with clinically significant post-traumatic stress symptoms. Participants were identified through symptom screening, assessed using the Clinician-Administered PTSD Scale for Diagnostic and Statistical Manual-5, randomised to eye movement desensitisation and reprocessing (EMDR) plus treatment as usual or treatment as usual alone, and followed up at 12 months. The study showed that critical illness survivors can be identified, consented, randomised, treated, and retained within this pathway, with encouraging therapy uptake and adherence, no treatment-related serious adverse events identified through trial monitoring, and broad qualitative acceptability among participants and therapists. Exploratory clinician-rated PTSD outcome estimates were hypothesis-generating and provide parameters for future trial planning, but should not be interpreted as evidence of effectiveness.

**Implications of all the available evidence**

The available evidence supports progression to a definitive multicentre trial to test the clinical and cost-effectiveness of trauma-focused psychological therapy for ICU survivors with clinically significant post-traumatic stress symptoms. Future trials should use sensitive but clearly trauma-anchored screening, clinician-rated and patient-reported outcomes, robust fidelity monitoring, prospective mapping of treatment as usual, and embedded process evaluation. Consistent with Medical Research Council guidance for complex interventions, EMDR after critical illness should be evaluated as an intervention whose effects may depend on delivery context, therapist expertise, fidelity, dose, usual-care variation, and participant characteristics. Future studies should also account for equity of access, patient preference, shared decision making, and potential mechanisms of response.

## Introduction

Post-traumatic stress disorder (PTSD) is characterised by intrusive re-experiencing, avoidance, negative alterations in cognition and mood, and hyperarousal following exposure to traumatic events. Recognition that severe medical illness can constitute traumatic stressors has expanded substantially, a shift formalised in the Diagnostic and Statistical Manual of Mental Disorders, Fifth Edition, Text Revision (DSM-5-TR),^1^ with important implications for survivors of critical illness.

For some survivors, critical illness involves perceived threat to life, invasive life-support technologies, severe physiological distress, impaired communication and loss of autonomy. These experiences may be accompanied by panic, dyspnoea, hallucinations, persecutory delusions, and fragmented or distorted memories arising in the context of delirium, critical illness and deep sedation.^2^ These features closely align with established PTSD risk factors.^3^ Approximately 20–30% of critical illness survivors experience clinically significant post-traumatic stress symptoms,^4^ representing a substantial population-level burden given that around 200,000 people are discharged alive from intensive care units each year in the UK alone.^5^

Traumatic stress after critical illness is associated with reduced health-related quality of life, impaired functional recovery, delayed return to work, increased healthcare utilisation, and elevated risk of suicidality.^6^^,^^7^ Recognition can be difficult because survivors may present months or years after discharge with non-specific psychological distress, somatic symptoms, or functional decline rather than explicit trauma narratives.^8^ Symptoms also commonly co-exist with depression, anxiety, cognitive impairment, and physical disability, complicating assessment and management.^9^

Despite increasing recognition of psychological morbidity after critical illness, evidence for effective psychological interventions following critical illness remains limited. Follow-up clinics, patient diaries, and multidisciplinary rehabilitation services have been evaluated, but implementation is inconsistent and systematic reviews report uncertain or inconsistent effects on longer-term mental health and quality-of-life outcomes.^10^^,^^11^ More recently, the PICTURE trial showed that a brief general practitioner-led narrative exposure intervention was feasible and reduced self-reported PTSD symptoms, although the effect was smaller than the predefined minimal clinically important difference.^12^ This supports trauma-informed approaches after intensive care, but uncertainty remains about specialist trauma-focused therapies, staged symptom-screening pathways, clinician-rated PTSD outcomes, and how interventions should be targeted. These uncertainties have prompted calls for more precise, needs-based models of mental health care following critical illness.^13^

Trauma-focused psychological therapies are evidence-based treatments for PTSD. Eye movement desensitisation and reprocessing (EMDR) is supported by systematic reviews and meta-analyses^14^ and recommended by international guidelines alongside trauma-focused cognitive behavioural therapies.^15^^,^^16^ However, evidence for EMDR in medical-event-related traumatic stress is smaller and more heterogeneous than for PTSD more broadly, and robust randomised evidence in survivors of critical illness remains lacking.^17^

EMDR may be relevant to traumatic stress following critical illness because treatment involves structured recall of distressing material within a protocolised trauma-focused therapy.^18^ Critical care-related memories can be fragmented, sensory-dominated, and poorly contextualised, reflecting perceived threat, delirium, hypoxia, sedation, and severe physiological stress.^19^ However, EMDR mechanisms, including the contribution of eye movements or other bilateral stimulation, remain debated. Preliminary empirical data provide limited support for further evaluation.^20^ Early-phase randomised studies are therefore needed to establish whether EMDR can be delivered feasibly and acceptably to survivors of critical illness, to estimate recruitment, retention, adherence, safety, and outcome variability, and to inform the design of a definitive trial, consistent with Medical Research Council guidance for complex interventions.^21^

We hypothesised that a staged pathway of symptom screening, clinician-rated PTSD assessment, randomisation, and EMDR delivery would be feasible and acceptable. The aim of this study was to assess the feasibility and acceptability of delivering EMDR to survivors of critical illness with clinically significant post-traumatic stress symptoms, and to generate exploratory clinical outcome estimates to inform a future definitive randomised controlled (RCT) trial.

## Methods

### Study design and participants

The EMERALD study was a mixed-methods, randomised, single-blind, parallel group-controlled, feasibility trial comparing EMDR with treatment as usual (TAU) in survivors of critical illness with post-traumatic symptoms. Recruitment took place across three UK National Health Service (NHS) trusts (University Hospital Southampton NHS Foundation Trust, Royal Bournemouth Hospital, and Poole General Hospital). The first participant consented on Feb 20, 2023, and final 12-month follow-up was completed on May 14, 2025.

The study adhered to CONSORT 2010 and SPIRIT 2013 guidance, was consistent with Medical Research Council complex intervention guidance^21^ and incorporated an embedded qualitative process evaluation using the Theoretical Framework of Acceptability (TFA).^22^ Patients and members of the public with lived experience of critical illness contributed to study design, participant-facing materials, and interpretation and reporting of findings through regular patient and public involvement meetings.

Eligible participants were adult survivors (≥18 years) of an intensive care admission who had received critical care for more than 24 h at a participating site and had capacity to provide informed consent. Exclusion criteria included pre-existing psychotic disorder or dementia, traumatic brain injury, or not expected to survive to hospital discharge. Participants with previous trauma exposure or traumatic stress were not excluded.

Consecutive eligible survivors were approached before hospital discharge or within two months following discharge. After providing written informed consent, participants entered an observational cohort and completed baseline demographic and psychometric assessments. At 2–3 months following hospital discharge, participants were recontacted and those scoring ≥22 on the Impact of Event Scale–Revised (IES-R),^23^ indicating post-traumatic stress symptoms,^24^ were invited to consider providing additional consent to participate in the randomised trial. The IES-R was selected because it has been widely used in critical care survivorship research and was specified in the published protocol. The threshold of ≥22 was chosen to prioritise sensitivity within a feasibility screening pathway, rather than to establish a DSM-5 PTSD diagnosis. Screening did not establish whether all symptoms were attributable specifically to the critical care admission. PTSD symptoms were subsequently assessed using the Clinician-Administered PTSD Scale for DSM-5 (CAPS-5)^25^ interview prior to randomisation.

Demographic characteristics, including sex, gender identity, ethnicity, and relevant medical history, were self-reported. These data were collected to describe the sample and assess representativeness. A visual timeline of enrolment, consent, screening, randomisation, treatment delivery, and follow-up is provided in Supplementary Material Appendix 1.

### Ethics

Ethical approval was granted by the South Central–Hampshire A Research Ethics Committee, Health Research Authority, UK (IRAS 317291). The trial was prospectively registered (ClinicalTrials.gov NCT05591625) and was conducted in accordance with the principles of the Declaration of Helsinki and Good Clinical Practice. All participants provided written informed consent before enrolment in the observational cohort. Participants who met the symptom threshold for trial entry provided additional written informed consent before randomisation. The protocol manuscript has been published previously.^26^

### Randomisation and masking

Participants meeting the trial threshold for clinically significant post-traumatic stress symptoms were randomised 1:1 to EMDR plus TAU or TAU alone. Randomisation was conducted independently using a secure web-based system by Sealed Envelope (London, UK) without stratification or minimisation. Randomisation was implemented by the practice manager at the tertiary NHS psychological therapy service, who was independent of the Chief Investigator and outcome assessor. Allocation concealment was maintained until eligibility confirmation, completion of baseline assessments, and entry into the randomisation system. The practice manager notified participants of their treatment allocation and, for participants allocated to EMDR, initiated referral into the psychological therapy pathway. Given the nature of the intervention, participants and therapists were not masked. CAPS-5 outcome assessors and quantitative analysts remained blinded to allocation until database lock. No instances of inadvertent unblinding were identified.

### Procedures

EMDR was delivered within a tertiary NHS psychological therapy service by EMDR Europe-accredited psychological therapists with specialist trauma experience, under the supervision of a consultant clinical psychologist accredited in EMDR. Therapists received additional trial-specific training on ICU-related trauma, including fragmented and delusional memories, delirium-associated experiences, ongoing physical morbidity, fatigue, and adaptation of pacing for critical illness survivors. This training was delivered by the Chief Investigator and Consultant Clinical Psychologist as two 1-h virtual training sessions, supplemented by written protocol materials and monthly supervision.

The intervention followed the standard eight-phase EMDR protocol^18^: history taking and treatment planning; preparation and stabilisation; assessment of target memories, negative cognitions, subjective units of distress and validity of cognition; desensitisation using bilateral stimulation while holding the target memory in mind; installation of an adaptive positive cognition; body scan to identify residual somatic distress; closure; and re-evaluation at subsequent sessions. Bilateral stimulation was delivered using therapist-guided eye movements, tapping, or auditory stimulation, according to participant preference, clinical need, and delivery mode. For remote sessions, therapists used videoconferencing with self-tapping or visually guided eye movements where appropriate. On one occasion, a therapist used a wand to facilitate saccadic eye movements for a visually impaired participant.

Participants were offered up to 16 weekly sessions of approximately 1 h. This upper limit was chosen pragmatically to remain close to guideline-recommended EMDR treatment while allowing additional sessions where clinically indicated. NICE guidance recommends that EMDR for adults is typically delivered over 8–12 sessions, but allows more sessions for people with multiple traumas or greater clinical complexity. In this population, critical care-related PTSD frequently involved multiple distressing targets, including delirium-associated or fragmented memories, repeated experiences of perceived life threat, invasive treatment, loss of autonomy, and, in some cases, earlier traumatic experiences that emerged during formulation and were clinically relevant to treatment. Treatment therefore proceeded by identifying and processing individual trauma targets, with completion defined collaboratively when key targets had been processed and associated distress had reduced, typically to a level where subjective units of distress was 0–1. A score of 0 indicates no distress, while 10 represents maximum possible distress. The main intervention procedures are described above. Additional details, including adaptations for critical illness, supervision arrangements, and fidelity procedures are provided in the published protocol^26^ and in the TIDieR (Template for Intervention Description and Replication) checklist in Supplementary Material Appendix 2.

An early protocol amendment redirected EMDR delivery from routine NHS psychological therapy services to the tertiary trauma service to ensure treatment fidelity; this amendment was approved by the sponsor and ethics committee before further randomisation.

TAU reflected existing post-ICU recovery pathways broadly consistent with NICE guidance, with minor site-level variation.^27^ Usual care typically included ward-based post-ICU clinical assessment, structured telephone follow-up after discharge, and multidisciplinary ICU follow-up clinic review where ongoing needs were identified. Psychological screening or assessment could inform referral to psychology, primary care, NHS Talking Therapies or community mental health services according to local pathways and clinical need. Some patients could receive brief psychological support within critical care follow-up services, and visits or patient diaries were offered where available. Individual-level psychological contacts, informal support, and community mental health contacts outside the trial were not systematically captured.

### Outcomes

The primary objective of the study was to assess the feasibility and acceptability of trial processes to participants and staff. The following feasibility targets were prespecified in the trial protocol: an average recruitment of 10 participants per month across the three participating sites, ≥30% of eligible patients approached would consent to participate, ≥75% of participants allocated to EMDR would complete at least 75% of planned therapy sessions, and ≥75% of randomly assigned participants would complete follow-up assessment at 12 months.

Acceptability of the intervention and trial procedures was evaluated through a qualitative sub-study guided by the TFA.^22^ Semi-structured interviews were conducted with a purposive sample of intervention recipients and all therapists. Interviews were analysed using a deductive framework approach; participant and therapist interview guides and detailed qualitative methods are reported in the protocol paper.^26^

Exploratory clinical outcomes were included to generate preliminary estimates of variability for a future definitive trial. The primary exploratory outcome was change in clinician-rated PTSD symptom severity measured by the CAPS-5 from 3 months (pre-intervention) to 12 months post-hospital discharge. All CAPS-5 assessments were conducted by a trained assessor (AB), who was blind to group allocation and has completed accredited training and supervised practice. The 12-month follow-up was anchored to hospital discharge rather than the critical care admission or discharge, to reflect the start of post-hospital recovery and align follow-up timing across participants entering the post-discharge pathway.

Secondary exploratory outcomes included self-reported PTSD symptoms on the Impact of Events Scale-Revised (IES-R), depression using the Patient Health Questionnaire-9 (PHQ-9), anxiety using the Generalised Anxiety Disorder-7 (GAD-7), and health-related quality of life EuroQol 5 Dimensions 5 Levels (EQ-5D-5L), assessed at baseline, 3 months, and 12 months following hospital discharge. Further details regarding secondary outcomes are provided in Supplementary Material Appendix 3.

Serious adverse events were defined as death, life-threatening events, hospitalisation, prolongation of hospitalisation, persistent disability, or other medical or psychologically significant event. Non-serious adverse events included any untoward medical or psychological occurrence during trial participation. Events were identified through participant, therapist or research-team report, and medical-record review where applicable. Psychological risk was monitored during therapy and outcome assessments, including PHQ-9 item 9. Suicidal ideation, symptom worsening, or therapist concern would trigger clinical risk assessment and escalation through the treating NHS psychological therapy service.

### Statistics

Because this was a feasibility trial, no formal sample size calculation was undertaken to detect clinical effectiveness. We aimed to recruit 160 participants to the observational cohort, anticipating that 20–25% would have clinically significant post-traumatic stress symptoms and that approximately 40 would enter the randomised comparison, providing sufficient precision to estimate feasibility parameters and outcome variability for a future definitive trial.^28^

Analyses followed best-practice guidance for feasibility trials and were conducted according to a prespecified analysis plan. Descriptive statistics summarised feasibility metrics, baseline characteristics, and outcomes. Feasibility parameters are reported as proportions with exact 95% CIs.

All analyses were conducted according to the intention-to-treat principle, including all participants as randomised, irrespective of intervention adherence. Change scores were summarised using appropriate measures of central tendency and dispersion. Because a baseline imbalance in CAPS-5 total score was observed, an exploratory baseline-adjusted analysis of 12-month CAPS-5 total score was undertaken using analysis of covariance, with treatment group and baseline CAPS-5 total score included as covariates. Clinical outcome analyses were exploratory and are reported using effect estimates and 95% confidence intervals to inform future trial design; p values, where presented, should not be interpreted as confirmatory tests of treatment effectiveness.

Qualitative interview data were analysed using a deductive framework approach guided by the TFA, with transcripts coded to the seven pre-defined constructs.^22^ Analytic procedures and reflexive considerations are described in detail in the published protocol.^26^

### Role of the funding source

Andrew Bates was funded by a National Institute for Health and Social Care Research Clinical Doctoral Research Fellowship (grant number: NIHR302160). The funder of the study had no role in study design, data collection, data analysis, data interpretation, or writing of this report.

## Results

**Fig. 1:**
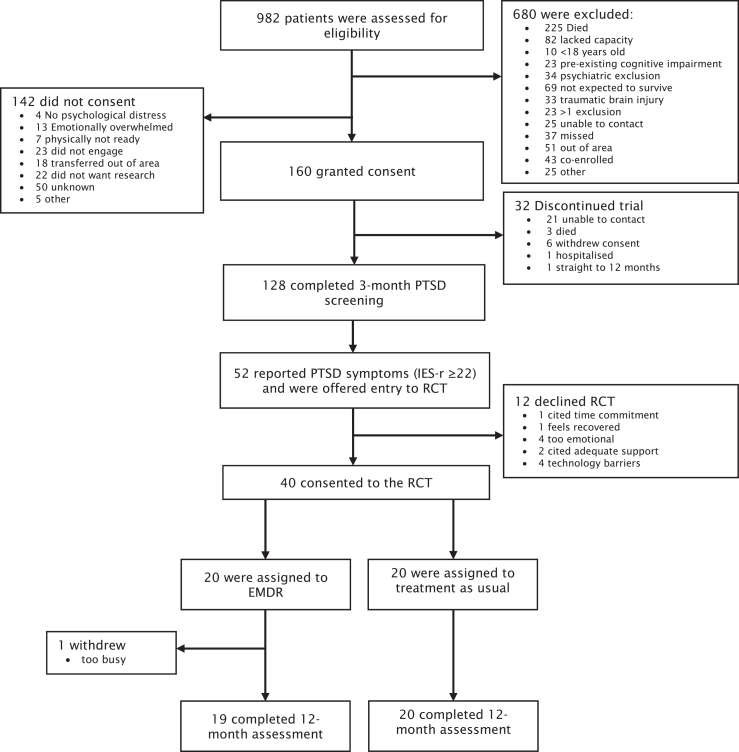
**Study profile.** Flow diagram showing eligibility assessment, exclusions, consent to the observational cohort, 3-month post-traumatic stress disorder screening, randomisation, allocation, withdrawal, and 12-month follow-up. EMDR = eye movement desensitisation and reprocessing; IES-R = Impact of Event Scale–Revised; PTSD = post-traumatic stress disorder; RCT = randomised controlled trial.

Between Feb 8, 2023, and May 14, 2024, 982 ICU survivors were screened, 302 were eligible, and 160 consented to the observational cohort, exceeding our pre-specified recruitment target. Of these, 128/160 (80%) completed 3-month PTSD symptom screening, 52/128 (41%) met the IES-R threshold for trial entry and 40/52 (77%; 95% CI 63.0–87.7) consented to randomisation: 20 to EMDR plus treatment as usual and 20 to treatment as usual alone. One participant allocated to EMDR withdrew before 12-month follow-up, and 39/40 (98%; 95% CI 86.8–99.9) of the 40 randomised participants completed the 12-month CAPS-5 assessment. Reasons for exclusion, non-consent, non-randomisation and withdrawal are shown in the Fig. 1.

Of the 20 participants allocated to EMDR, 18 (90%; 95% CI 68.3–98.8) initiated treatment. Across all EMDR-allocated participants, the mean number of sessions attended was 10.4 (SD 7.0). 15 (75%) of 20 completed a full therapeutic course, defined as continuing EMDR until the agreed trauma targets had been processed and Subjective Units of Distress had reduced to 0–1, meeting the predefined adherence feasibility threshold. Two participants discontinued after two and three sessions, respectively, because of physical health deterioration, and one withdrew after a single session owing to time constraints. Among those who completed treatment, 12 participants received ten or more sessions, and seven completed the full 16-session protocol.

Referral pathway fidelity was compromised early in the trial. Of the first four participants randomised to EMDR, only one received treatment within Community Mental Health Teams (CMHT), and subsequently received 23 sessions, which was determined to be a protocol deviation. The remaining three were declined treatment despite meeting eligibility criteria. Following sponsor and ethical approval, referrals were redirected to a tertiary-level NHS psychological therapy service with EMDR expertise which provided treatment to the initially declined participants and all subsequently randomised participants.

No treatment-related serious adverse events were identified. No participants required emergency unblinding or withdrawal from trial procedures because of psychological deterioration. No participant required escalation to crisis or emergency mental health services during EMDR. Qualitative interviews indicated that EMDR and trial procedures were broadly acceptable to participants and therapists, but acceptability was not uniform or uncomplicated. Some participants described initial scepticism about EMDR, particularly the unfamiliar bilateral-stimulation techniques, while others found aspects of reprocessing repetitive, emotionally tiring, or distressing. A minority avoided the most difficult material or found it hard to sustain engagement when physical recovery, work, or other health demands competed with therapy. Therapists also described additional emotional labour when working with therapy-naïve ICU survivors, uncertainty about how to adapt EMDR for fragmented or delusional ICU memories, and some discomfort about randomisation to treatment as usual.

These concerns did not generally prevent engagement, and many participants described EMDR as demanding but worthwhile once therapeutic trust and intervention coherence developed. Across TFA constructs, acceptability appeared to depend less on the technique alone than on pacing, therapeutic relationship, flexibility of delivery, and access to specialist supervision. A summary of TFA-based findings is provided in the Panel 1, with construct-level tables and exemplar quotes in Supplementary Material Appendices 4 and 5. A joint display integrating feasibility, quantitative outcome, and qualitative acceptability findings by TFA construct is provided in Appendix 7. Baseline demographic and clinical characteristics were broadly comparable between groups, although apparent imbalances should be interpreted cautiously given the small sample (Table 1). The cohort had a median age of 59.5 years (IQR 52–66), balanced sex distribution, predominantly White ethnicity, and modest over-representation from less deprived areas. Some clinical characteristics differed numerically between groups, including diabetes, ischaemic heart disease, and peripheral vascular disease. The study was not powered to assess the prognostic importance of these differences, and no additional covariate-adjusted analyses were undertaken. Baseline CAPS-5 severity was also higher in the EMDR group; therefore, exploratory baseline-adjusted analyses were undertaken for clinician-rated PTSD outcomes.

Pane l **Summary of qualitative acceptability findings**

**Affective attitude**

Participants and therapists viewed EMDR positively overall, describing the intervention as emotionally demanding but worthwhile. Initial scepticism about EMDR techniques tended to diminish as therapeutic rapport strengthened, with most participants reporting increased confidence and engagement over time.

**Burden**

The emotional effort required to revisit ICU-related trauma was challenging and, for some, associated with fatigue, anticipatory dread, or distress during sessions. Practical burdens were less prominent, although competing health needs and appointments affected engagement for a minority. Participants reported no meaningful difference in burden between online and face-to-face delivery, and several valued having the choice of format, which further supported engagement.

**Intervention coherence**

Most participants reported a clear understanding of the rationale for EMDR and how it related to their ICU experiences. Despite some initial scepticism, many described a progression towards EMDR helping to organise intrusive or fragmented memories, reinforcing a sense of coherence between the intervention and their recovery needs.

**Ethicality**

EMDR was viewed as appropriate and aligned with participants’ values and expectations for psychological recovery after critical illness. Some also mentioned an altruistic motive for undertaking research. Therapists also perceived the intervention as ethically appropriate and clinically congruent with trauma-focused work.

**Opportunity costs**

Opportunity costs were generally limited, but some participants avoided the most distressing material or had to balance therapy against physical recovery, hospital appointments, work, or caregiving demands.

**Perceived effectiveness**

Many participants and therapists perceived symptomatic and functional gains, but some described partial improvement, ongoing uncertainty about what had happened in ICU, or persistent difficulty engaging with more complex trauma material.

**Self-efficacy**

Participants generally gained confidence in their ability to engage with emotionally intense sessions as therapy progressed. Therapists expressed high confidence in delivering EMDR within the trial protocol and viewed the intervention as feasible within specialist psychological services.

**Table 1:** Baseline demographic and clinical characteristics.

Baseline demographics	TAU (n = 20)	EMDR (n = 20)
Age (years)	58.0 (50.0–66.0)	59.5 (53.5–66.0)
Sex		
Male	8 (40%)	11 (55%)
Female	12 (60%)	9 (45%)
Ethnicity		
White British	18 (90%)	19 (95%)
Other	2 (10%)	1 (5%)
Employment status		
Full-time	6 (30%)	7 (35%)
Part-time	3 (15%)	2 (10%)
Unemployed	3 (15%)	1 (5%)
Retired	6 (30%)	7 (35%)
Other	2 (10%)	3 (15%)
Living arrangement		
Independent	20 (100%)	19 (95%)
At home with care	0 (0%)	1 (5%)
Index of Multiple Deprivation decile	7.0 (4.5–8.5)	5.0 (2.5–8.0)
APACHE II score	17.5 (13.0–19.0)	19.0 (15.0–21.0)
Level 3 care (days)	5.0 (3.0–11.5)	4.5 (3.0–8.0)
Critical care LOS (days)	12.0 (5.0–17.5)	7.5 (5.0–10.0)
Hospital LOS (days)	18.0 (14.5–37.0)	14.5 (10.0–26.0)
Inotrope use (days)	4.0 (2.5–6.0)	4.0 (2.0–8.0)
Sedative use (days)	4.5 (3.0–8.5)	4.0 (3.0–7.0)
Delirium positive (days)	1 (0–1)	0 (0–3)
Benzodiazepine use (days)	0 (0–1)	0 (0–1)
COPD	4 (20%)	3 (15%)
Asthma	2 (10%)	3 (15%)
Ischaemic heart disease	0 (0%)	3 (15%)
Chronic liver disease	1 (5%)	0 (0%)
Diabetes	4 (20%)	8 (40%)
Chronic renal failure	1 (5%)	1 (5%)
Hypertension	8 (40%)	10 (50%)
Peripheral vascular disease	5 (25%)	0 (0%)
CVA/cerebral haemorrhage	0 (0%)	1 (5%)
Mental health diagnosis	3 (15%)	4 (20%)

Data are n (%) for categorical variables and median (IQR) for continuous variables. APACHE II = Acute Physiology and Chronic Health Evaluation II; LOS = length of stay; IMD = Index of Multiple Deprivation. Higher IMD deciles indicate greater socioeconomic deprivation. COPD = Chronic obstructive pulmonary disease; CVA = Cerebrovascular accident.

Pre-randomisation psychological symptom scores are shown in Table 2. Median CAPS-5 total score was higher in participants allocated to EMDR than treatment as usual, indicating an imbalance in clinician-rated PTSD severity at RCT baseline. Self-reported PTSD, anxiety and depressive symptoms were more similar across groups. Because of the CAPS-5 imbalance, baseline-adjusted exploratory analyses were undertaken for clinician-rated PTSD outcomes. Clinical outcome analyses were exploratory and intended to estimate outcome distributions, variability and direction of effect for a future definitive trial, rather than to test treatment effectiveness. Clinician-rated PTSD symptom severity decreased more in participants allocated to EMDR than in those allocated to treatment as usual (Table 3). Mean CAPS-5 total score change was −15.6 (SD 12.5) in the EMDR group and −1.6 (SD 11.8) in the treatment-as-usual group. The unadjusted between-group difference in change was −14.1 points (95% CI −22.0 to −6.2). After adjustment for baseline CAPS-5 total score, the estimated between-group difference was −11.1 points (95% CI −19.0 to −3.2) (see Supplementary Material Appendix 6 for further detail). A similar pattern was observed for CAPS-5 diagnostic criterion count. These estimates should be interpreted cautiously because the trial was not powered to detect clinical effectiveness, the sample was small, and multiple clinical outcomes were examined. CAPS-5 diagnostic status is reported descriptively in Table 4. At RCT baseline, 11 of 20 participants in the treatment-as-usual group and 13 of 20 in the EMDR group met full CAPS-5 diagnostic criteria for PTSD. At 12 months, PTSD diagnosis was present in 8 of 20 participants in the treatment-as-usual group and 4 of 19 in the EMDR group. Diagnostic trajectories were numerically consistent with the clinician-rated symptom findings, but these categorical results are based on small numbers and should be regarded as hypothesis-generating. Patient-reported PTSD, anxiety, depression and health-related quality of life generally improved between 3 and 12 months in both groups (Table 5). Reductions in IES-R scores were numerically larger in the EMDR group, but the between-group estimate was imprecise. Changes in GAD-7, PHQ-9 and EQ-5D-5L index scores were similar across groups, with confidence intervals compatible with little or no between-group difference. These outcomes are reported descriptively to inform outcome selection and variance estimates for a future definitive trial.

**Table 2:** Baseline psychological symptom scores before randomisation.

Variable	TAU (n = 20)	EMDR (n = 20)
CAPS-5 total	23.5 (18.5–30.0)	31.0 (21.0–36.0)
IES-R	39.0 (31.5–51.5)	40.5 (28.5–53.0)
GAD-7	9.5 (5.5–14.5)	10.0 (5.5–16.5)
PHQ-9	15.0 (6.0–18.5)	11.0 (8.5–13.5)

Data are median (IQR). CAPS-5 = Clinician-Administered PTSD Scale for DSM-5; IES-R = Impact of Event Scale–Revised; GAD-7 = Generalised Anxiety Disorder–7 questionnaire; PHQ-9 = Patient Health Questionnaire–9. Higher scores indicate greater symptom severity.

**Table 3:** Changes in clinician-rated PTSD severity from baseline to 12 months.

Outcome	TAU (n = 20)	EMDR (n = 19)	Unadjusted between group difference (95% CI)	p value	Adjusted between group difference (95% CI)	p value
CAPS-5 total score	−1.55 (11.84)	−15.63 (12.46)	−14.08 (−21.96, −6.20)	0.001	−11.08 (−18.97, −3.19)	0.007
CAPS-5 criterion count	−0.95 (5.71)	−7.74 (5.77)	−6.79 (−10.51, −3.06)	0.001	−4.68 (−8.17, −1.19)	0.010

Data are mean change (SD). Negative values indicate symptom improvement. Between-group differences are mean differences (EMDR minus TAU) with 95% CIs. Adjusted analyses control for baseline CAPS-5 scores. TAU = Treatment as usual; EMDR = Eye movement desensitisation and reprocessing; CAPS-5 = Clinician-Administered PTSD Scale for DSM-5; CI = Confidence interval.

**Table 4:** Clinician diagnosed PTSD at 3 and 12 months.

	TAU n = 20	EMDR n = 19
PTSD diagnosis (CAPS-5)		
PTSD at 3 months	11 (55%)	13 (65%)
PTSD at 12 months	8 (40%)	4 (21%)
PTSD status trajectory		
PTSD at both timepoints	7 (35%)	3 (16%)
No PTSD at both timepoints	8 (40%)	5 (26%)
Changed from PTSD to no PTSD	4 (20%)	10 (53%)
Changed from no PTSD to PTSD	1 (5%)	1 (5%)

Data are n (%). PTSD diagnosis was determined using the Clinician-Administered PTSD Scale for DSM-5 (CAPS-5). Percentages at 12 months are based on 20 participants in the TAU group and 19 in the EMDR group. PTSD trajectory categories reflect diagnostic status at 3 and 12 months.

**Table 5:** Patient-reported outcome measures at 3 and 12 months.

Outcome measure	TAU (n = 20)	EMDR (n = 19)	Between-group difference (95% CI)	p value
IES-R				
IES-R at 3 months	40.8 (12.1)	41.7 (14.8)	–	–
IES-R at 12 months	31.4 (14.8)	23.5 (19.3)	–	–
**Change in IES-R (12 m – 3 m)**	−9.5 (19.8)	−18.3 (19.2)	−8.9 (−21.8 to 4.0)	0.17
GAD-7				
GAD-7 at 3 months	10.0 (5.0)	10.3 (6.5)	–	–
GAD-7 at 12 months	8.9 (5.2)	7.9 (6.4)	–	–
**Change in GAD-7 (12 m – 3 m)**	−1.1 (6.8)	−2.4 (7.2)	−1.3 (−6.0 to 3.3)	0.56
PHQ-9				
PHQ-9 at 3 months	13.7 (7.4)	11.8 (5.0)	–	–
PHQ-9 at 12 months	9.2 (6.8)	7.6 (5.7)	–	–
**Change in PHQ-9 (12 m – 3 m)**	−4.5 (8.0)	−4.7 (6.3)	−0.2 (−5.0 to 4.6)	0.93
EQ-5D-5L index				
EQ-5D-5L index at 3 months	0.574 (0.255)	0.502 (0.244)	–	–
EQ-5D-5L index at 12 months	0.722 (0.202)	0.625 (0.224)	–	–
**Change in EQ-5D-5L index (12 m – 3 m)**	0.148 (0.252)	0.145 (0.229)	−0.003 (−0.162 to 0.156)	0.97
EQ-5D VAS				
VAS at 3 months	50 (35–65)^a^	52 (40–64.5)^a^	–	–
VAS at 12 months	65.5 (54.5–77.5)^a^	60 (50–71)^a^	–	0.34

Values shown as mean (SD) unless otherwise specified. Negative change values indicate symptom improvement for IES-R, GAD-7, and PHQ-9. EQ-5D-5L index scores derived using the UK 5 L valuation study (Devlin et al., 2018). Between-group differences are unadjusted mean differences in change scores with 95% CIs. The study was not powered to detect between-group differences in PROMs.

a VAS values reported as median (IQR).

## Discussion

The EMERALD study shows that a trauma-focused EMDR pathway for survivors of critical illness with clinically significant post-traumatic stress symptoms can be delivered within a multicentre randomised feasibility trial. We identified eligible survivors, used staged symptom screening and clinician assessment, randomised participants, and achieved high retention to 12-month follow-up. Intervention uptake and adherence were encouraging, no treatment-related serious adverse events were identified within trial monitoring, and qualitative data suggested broad acceptability. The trial was not powered to test effectiveness but generated exploratory clinician-rated PTSD outcome estimates to inform a definitive multicentre evaluation.

The feasibility outcomes address an important gap in post-critical illness psychological care. Psychological morbidity after intensive care is well recognised, yet trauma-focused interventions are not routinely embedded within recovery pathways.^29^ Systematic reviews describe heterogeneous follow-up models, variable uptake, and uncertain effects on PTSD and related outcomes.^10^ EMERALD suggests that, when a trauma-focused intervention is clearly specified, supervised, and embedded within a structured screening pathway, many survivors are willing to undergo assessment, initiate therapy, and remain engaged to long-term follow-up. These findings do not establish scalability, but they identify delivery conditions for a definitive trial to reproduce and test.

Several design features strengthen the feasibility findings. The staged recruitment moved from broad ICU survivor enrolment to symptom screening at 2–3 months after discharge, followed by clinician-rated PTSD assessment before randomisation.^16^^,^^27^ This targeted a clinically relevant subgroup while avoiding trauma-focused therapy for all ICU survivors irrespective of need, consistent with calls for more personalised recovery models.^13^ The mixed-methods design integrated recruitment, uptake, adherence, retention, exploratory clinical outcomes, and qualitative acceptability data supporting future trial optimisation without establishing intervention effects or mechanisms.^21^

The qualitative findings suggested that EMDR was broadly acceptable, but not uniformly so. Participants described EMDR as emotionally demanding; some reported scepticism, distress during reprocessing, fatigue, difficulty with specific components, or avoidance of the most difficult material. Therapists described emotional labour when working with therapy-naïve critical care survivors, uncertainty when adapting EMDR to fragmented or delusional memories, and tension between protocol fidelity and clinical responsiveness. These divergent cases strengthen the feasibility interpretation by showing that engagement depended on pacing, therapeutic alliance, flexible delivery, and specialist supervision, not simply availability of EMDR.^30^ We did not include a validated quantitative acceptability measure to support triangulation; future trials should include one. Integrated mixed-methods findings are shown in Appendix 7.

The exploratory clinician-rated PTSD findings are clinically relevant but require cautious interpretation. CAPS-5 symptom severity decreased more in participants allocated to EMDR than in those receiving treatment as usual, including after adjustment for baseline CAPS-5 severity. However, the sample was small, baseline imbalance was present, multiple clinical outcomes were examined, and early-phase trials can overestimate treatment effects. Other clinical characteristics, including diabetes, ischaemic heart disease, and peripheral vascular disease, also differed numerically and may have influenced recovery trajectories. The estimates should therefore be viewed as hypothesis-generating and useful for planning a conservatively powered definitive trial, rather than as confirmatory evidence of effectiveness. Future sample size assumptions should be informed by clinical importance, patient and public involvement, retention, adherence, observed variability, baseline to follow-up correlation, and allowance for attrition, site-level variation, therapist effects, and potential effect-size inflation.

The flexible number of EMDR sessions is another important consideration. Offering up to 16 sessions reflected target-based EMDR treatment and the anticipated complexity of critical illness-related trauma, including fragmented or delirium-associated memories, repeated perceived life threat, invasive treatment, loss of autonomy, and earlier traumatic experiences that emerged during formulation. This was appropriate for a feasibility study, but it limits interpretation of dose–response effects. EMERALD was not designed to determine the minimum effective dose, optimal treatment duration, or whether outcomes varied by treatment exposure. A definitive trial should pre-specify dose ranges, collect structured session-level data, and examine dose, fidelity, and clinical response within the process evaluation.

Intervention fidelity is a key limitation. Fidelity was supported through therapist accreditation, supervision, peer support, and session records, but independent session-level fidelity rating using the EMDR Fidelity Rating Scale was not implemented. Treatment drift, variation in delivery, therapist effects, and the consistency of ICU-specific adaptations could therefore not be directly assessed. This matters because EMDR after critical illness may require adaptation for delirium-related memories, cognitive fatigue, physical morbidity, and complex trauma histories. A definitive trial should include prospective fidelity procedures, including session recording or alternative observation, independent expert rating, supervision logs, therapist feedback, and pre-specified documentation of adaptations, to support consistent delivery and distinguish intervention effects from therapist-level variation.^22^

Follow-up was scheduled 12-months after hospital discharge, but participants varied in ICU and hospital length of stay, time from ICU discharge to hospital discharge, and time from screening to treatment initiation. This may have introduced variability in time since the critical illness and duration of post-treatment follow-up. Future trials should prospectively report time from ICU admission, ICU discharge, hospital discharge, randomisation, treatment initiation, and treatment completion to each outcome assessment.

Clinician-rated and patient-reported outcomes diverged. Patient-reported PTSD, anxiety, depression, and health-related quality of life improved in both groups, with less between-group separation than for clinician-rated PTSD, consistent with other studies.^10^ Structured clinician interviews may better anchor symptoms to the critical care-related index trauma, particularly where memories are fragmented, sensory-dominated, or shaped by delirium and sedation.^19^ Alternatively, trauma-specific improvement may precede broader changes in psychological distress or quality of life, which are also influenced by physical recovery, social functioning, comorbidity, and ongoing healthcare needs.^31^

CAPS-5 assessments were conducted by a blinded assessor; however, assessment-related bias cannot be excluded in a small feasibility trial. Future trials should strengthen outcome assessment through standardised rater training, masking procedures, documentation of unmasking, and inter-rater reliability checks, while retaining complementary patient-reported outcomes.^32^

Interpretation also depends on the nature of treatment as usual and delivery configuration. TAU reflected real-world post-critical care recovery pathways broadly consistent with NICE guidance,^27^ enhancing external validity but introducing heterogeneity in access to psychological services. TAU was described at pathway level, but individual-level psychological contacts, co-interventions, informal support, and community mental health contacts were not systematically captured, limiting assessment of contamination and usual-care exposure. Prior studies demonstrate that variation in usual care can act as an uncontrolled effect modifier.^10^ Early referral through routine community mental health pathways also proved unreliable, with some participants declined despite meeting trial eligibility criteria. Redirection to a tertiary NHS psychological therapy service improved delivery but limits conclusions about whether similar uptake, adherence, and fidelity could be achieved through generic community services. A definitive trial should prospectively map usual care, record psychological and recovery-related contacts, and test a sustainable delivery model, such as specialist trauma services, critical care-integrated psychology, or a hybrid pathway.^21^

Generalisability and equity also require caution. The sample was small, recruited from three UK NHS hospitals, predominantly White, and modestly over-represented by participants from less deprived areas.^5^ These factors limit external validity because access to psychological therapies and recovery after critical illness are shaped by structural determinants of health.^33^^,^^34^ Future trials should maximise ethnic and socioeconomic diversity through site selection, adapted materials where needed, flexible consent and follow-up procedures, remote and face-to-face options, and monitoring of recruitment, retention, treatment uptake, and missing data by ethnicity, deprivation, sex, gender, and other relevant characteristics. Trial design should consider stratification or minimisation by site and deprivation, and pre-specified analyses of differential engagement and response. Because access to trauma-focused therapy is central to scalability, future trials should evaluate proactive screening following critical illness, embedded referral pathways, remote delivery, flexible appointments, and links between critical care, primary care, and specialist psychological therapy services.

The screening strategy also has implications for interpretation. EMERALD used the IES-R threshold of ≥22 to prioritise sensitivity, an approach supported by patient and public contributors who preferred a symptom-based trial entry pathway over one restricted to clinician-diagnosed PTSD. However, the IES-R predates DSM-5, was not explicitly anchored to the critical care admission during screening, and is not equivalent to a DSM-5 PTSD diagnosis. The randomised sample therefore included participants with clinically significant post-traumatic stress symptoms, not all of whom met full CAPS-5 diagnostic criteria. This improves relevance to symptom-based post-ICU care, where distress may be important below diagnostic threshold, but complicates interpretation of diagnostic change. A definitive trial should retain staged screening while evaluating DSM-5-aligned tools such as the PCL-5, alternative thresholds, and concordance with clinician-rated PTSD severity.

Finally, mechanisms of EMDR were not tested. EMDR can be conceptualised as a trauma-focused therapy involving exposure to traumatic material alongside bilateral stimulation, but the specific contribution of eye movements or other bilateral stimulation remains debated. EMERALD cannot determine whether observed changes were mediated by adaptive memory processing, exposure, therapeutic alliance, expectancy, structured clinical contact, or other non-specific factors. Future trials should evaluate EMDR after critical illness as a complex intervention,^21^ specify a programme theory, and include process evaluation of fidelity, dose, adaptations, therapeutic alliance, expectancy, therapist effects, usual-care context, and participant characteristics. Mechanistic analyses should test candidate mediators and moderators, including baseline PTSD severity, trauma complexity, delirium-related memories, prior mental health history, physical recovery, and treatment dose.

In conclusion, EMERALD demonstrates the feasibility and broad acceptability of delivering targeted trauma-focused EMDR to critical care survivors with clinically significant post-traumatic stress symptoms within a randomised trial pathway. The study provides evidence of retention, treatment uptake, and exploratory clinician-rated outcome estimates, while identifying unresolved uncertainties around effectiveness, mechanisms, fidelity, dose, usual-care heterogeneity, screening, equity, delivery configuration, and subgroup response. These findings justify a definitive multicentre trial, but effectiveness, cost-effectiveness, safety profile, scalability, and mechanisms of action remain to be established.

## Contributors

AB, SR, and RC conceived the study, with input from NP, DSB, and MPWG. Methodology was developed by AB, RC, NP, DSB, and MPWG. Funding was acquired by AB, RC, NP, DSB, and MPWG. AB, RC, HG, SR, NP, DSB, and MPWG contributed to study investigation and delivery. AB, RC, HG, NP, DSB, and MPWG contributed to data curation. Formal analyses were undertaken by AB and HM, with input from RC and MPWG. AB and HM directly accessed and verified the underlying data. Project administration was led by AB and RC, with support from HG and MPWG. Software and data visualisation were undertaken by AB and HM. Supervision was provided by RC, SR, JH, NP, DSB, and MPWG. HS contributed patient and public involvement expertise, interpretation of findings, and manuscript review. AB drafted the original manuscript. All authors contributed to review and editing of the manuscript. All authors read and approved the final version of the manuscript. AB, with agreement from all authors, was responsible for the decision to submit the manuscript for publication.

## Data sharing statement

Deidentified clinical trial data may be requested by qualified researchers conducting independent scientific research. Requests will be considered following review and approval of a research proposal and analysis plan, and subject to a data access agreement in accordance with participant consent and applicable data protection regulations. Statistical code used for the quantitative analyses may be made available on reasonable request, subject to appropriate review and compatibility with data sharing and information governance requirements. For further information or to submit a request, please contact the corresponding author: a.bates@soton.ac.uk.

## Declaration of interests

AB reports support from a National Institute for Health and Care Research Clinical Doctoral Research Fellowship (NIHR302160), held by University Hospital Southampton NHS Foundation Trust, for the submitted work and is Nurse Professional Advisory Group member of the Intensive Care Society RC reports NIHR Biomedical Research Centre funding awards to her institution, independent of the submitted work; and unpaid roles as an Associate Panel Member for the UKRI Medical Research Council Experimental Medicine Panel and Council Member of the Critical Care Medicine Section, Royal Society of Medicine, London. NP reports grants from the National Institute for Health and Care Research paid to her institution, independent of the submitted work; unpaid participation on an advisory board for an NIHR study independent of this work; and unpaid roles as Chair of Trustees and Chair of the National Outreach Forum for a registered UK charity. DSB reports grants from the National Institute for Health and Care Research Health Technology Assessment programme and NIHR Translational Research Collaboration, independent of the submitted work; editorial honoraria from Elsevier and Wiley; and professional leadership roles as Past-President of the British Association for Psychopharmacology and Vice-President of the European College of Neuropsychopharmacology. MPWG reports unrestricted grant funding from Edwards Lifesciences, Becton Dickinson, and Pharmacosmos; honoraria for consulting or lecturing from Baxter, Mode Sensors, Analogue Solutions, Edwards Lifesciences, and Becton Dickinson; and funding from the NIHR Southampton Biomedical Research Centre and NIHR Senior Investigator Scheme, independent of the submitted work. HG, HM, HS, SR, and JH declare no competing interests.

## Appendix A Supplementary data

Appendices
