# Supplementary material for: Eye movement desensitisation and reprocessing for post-traumatic stress in survivors of critical illness (EMERALD): a mixed-methods, randomised, single-blind, parallel group-controlled, feasibility trial
Source: eClinicalMedicine. 2026 Jul 13;97:104082. doi: 10.1016/j.eclinm.2026.104082 (PMC13382324; doi:10.1016/j.eclinm.2026.104082)
Supplement: Appendices [file mmc1.docx]

**Supplementary material**

**Eye movement desensitisation and reprocessing for post-traumatic stress in survivors of critical illness: a mixed-methods, randomised, parallel group controlled feasibility trial**

Andrew Bates PhD^1,2^, Rebecca Cusack MD^1,2,3^, Hannah Golding MSc, Helen Moyses MSc^1^, Hazel Southam BA^4^, Sophie Rushbrook DClinPsych^5^, Julie Highfield DClinPsych^6^, Natalie Pattison PhD^7^, David S Baldwin DM^2,8,9^, Michael PW Grocott MD^1,2,3^

**Corresponding author**

Dr Andrew Bates

NIHR Southampton Biomedical Research Centre, University Hospital Southampton NHS Foundation Trust, Southampton, UK

Email: [a.bates@soton.ac.uk](mailto:a.bates@soton.ac.uk)

**Submitted to:** eClinicalMedicine

**Manuscript number:** eclinm-D-26-01043

**Contents**

Appendix 1. EMERALD participant timeline and study procedures

Appendix 2. EMDR treatment protocol reported according to TIDieR

Appendix 3. Additional information regarding exploratory clinical outcome measures

Appendix 4. Theoretical Framework of Acceptability (TFA): therapist perspectives on delivering EMDR

Appendix 5. Theoretical Framework of Acceptability (TFA): participant perspectives on receiving EMDR

Appendix 6. Exploratory baseline-adjusted analysis of clinician-rated traumatic stress severity

Appendix 7. Joint display of feasibility, quantitative outcomes, and qualitative acceptability findings structured by the Theoretical Framework of Acceptability (TFA)

References

**Appendix 1.** EMERALD participant timeline and study procedures

Screened for eligibility

ICU discharge – up to 3-months following hospital discharge

Part A consent

Baseline data collection (psychometric and QoL)

3-month data collection (psychometric and QoL)

IES-R ≥22

No

Yes

3-months following hospital discharge

RCT declined

Part B (RCT) consent

CAPS-5 Interview

Randomisation

Intervention period

3-12-months following hospital discharge

TAU

EMDR + TAU

CAPS-5 Interview

12-months following hospital discharge

12-month post hospital discharge data (psychometric and QoL)

**Figure S1:** Participants entered Part A after intensive care discharge and completed baseline demographic and patient-reported outcome assessments. At 2-3 months after hospital discharge, participants completed mental health screening. Participants scoring below the trial threshold <22 on the Impact of Events Scale-Revised (IES-R) continued in the observational cohort. Participants scoring ≥22 were invited to provide Part B consent. Decliners were invited to continue in the observational cohort. Those providing consent completed a CAPS-5 assessment and were randomised (1:1) to to EMDR plus treatment as usual (TAU) or TAU alone. Participants allocated to EMDR were offered up to 16 sessions between approximately 3 and 9 months after discharge. All randomised participants were invited to complete clinician-rated and patient-reported outcome assessment at 12 months after hospital discharge. CAPS-5=Clinician-Administered PTSD Scale for DSM-5; EMDR=eye movement desensitisation and reprocessing; IES-R=Impact of Event Scale–Revised; ICU=intensive care unit; PTSD=post-traumatic stress disorder; QoL=Quality of life;

**Appendix 2.** EMDR treatment protocol reported according to TIDieR (1).

### **1. Brief name**

Eye movement desensitisation and reprocessing (EMDR) for post-traumatic stress disorder following critical illness.

### **2. Why (rationale, theory, goal)**

EMDR is a trauma-focused psychological therapy hypothesised to alleviate post-traumatic stress symptoms by facilitating adaptive processing and integration of distressing traumatic memories. ICU-related trauma is frequently characterised by fragmented, sensory-dominated, and poorly contextualised memories arising in the context of delirium, sedation, and extreme physiological stress. Bilateral stimulation during recall of traumatic material is proposed to support memory reconsolidation and reduce the emotional salience of traumatic experiences. The goal of the intervention was to reduce clinician-diagnosed PTSD symptom severity in ICU survivors with established post-traumatic stress symptoms.

### **3. What (materials)**

No physical materials were required. For online delivery, NHS-approved videoconferencing platforms were used. Where necessary, participants were provided with a tablet device and internet dongle to enable access to remote sessions.

### **4. What (procedures)**

EMDR was delivered using the standardised eight-phase protocol:

1. **History taking and treatment planning:** identification of traumatic targets and formulation, including assessment of internal and external resources.
2. **Preparation:** establishment of therapeutic alliance, explanation of the EMDR process, and development of stabilisation strategies.
3. **Assessment:** identification of target memory, associated imagery, affect, negative cognition, and baseline ratings using Subjective Units of Distress (SUD) and Validity of Cognition (VOC).
4. **Desensitisation:** bilateral stimulation (eye movements, tapping, or auditory tones) while holding the target memory in mind, repeated until SUD reduced to 0–1.
5. **Installation:** strengthening of a positive cognition associated with the target memory.
6. **Body scan:** identification and processing of residual somatic distress.
7. **Closure:** restoration of emotional equilibrium at the end of each session.
8. **Re-evaluation:** review of previously processed material and identification of future targets at subsequent sessions.

Procedures were adapted for ICU-related trauma, including work with fragmented, delusional, or sensory memories, which could be processed without verbal narrative if preferred by the participant. The protocol allowed for pre-existing trauma, unrelated to the ICU stay, to be addressed during the sessions.

### **5. Who provided**

EMDR was delivered by experienced NHS psychological therapists accredited by EMDR Europe, with a minimum of three years’ post-qualification clinical experience. Therapists participated in regular peer support and received monthly supervision from an EMDR Europe-accredited Consultant Clinical Psychologist with specialist expertise in trauma-focused interventions.

### **6. How (mode of delivery)**

Sessions were delivered individually, either face-to-face in NHS psychological therapy clinics or remotely via secure NHS-approved videoconferencing, according to participant preference and accessibility needs.

### **7. Where**

Face-to-face sessions took place in NHS psychological therapy clinics within participating trusts. Remote sessions were conducted using Microsoft Teams™ or equivalent NHS-approved platforms. Participants unable to access either modality independently were supported with study-provided digital equipment.

### **8. When and how much**

Sessions were delivered approximately weekly, lasted up to 60 minutes, and participants were offered up to 16 sessions. Flexibility was allowed to account for ongoing medical treatment. Treatment duration and number of sessions were determined collaboratively between therapist and participant, with completion defined as resolution of identified trauma targets.

### **9. Tailoring**

The intervention was personalised in line with standard EMDR practice. Tailoring included selection and sequencing of trauma targets, pacing of reprocessing, and adaptation to cognitive, emotional, and physical sequelae of critical illness. ICU-specific adaptations included processing of delusional memories, repeated or cumulative ICU stressors, and pre-existing trauma where clinically indicated.

### **10. Modifications**

A protocol modification occurred early in the trial. Initially, participants randomised to EMDR were referred via NHS Improving Access to Psychological Therapies (IAPT) services. Following difficulties in accessing EMDR through this route and inconsistent adherence to the protocol, referrals were redirected to a tertiary-level psychological therapy service with established EMDR expertise. This amendment was approved by the sponsor and research ethics committee and ensured consistent delivery of the intervention for the remainder of the trial.

### **11. How well (planned fidelity)**

Treatment fidelity was supported through therapist accreditation requirements, structured supervision, and peer support. Adherence was monitored through therapist session records and supervision review. A representative sample of sessions was designed to be reviewed by an expert practitioner using the EMDR Fidelity Rating Scale. However, the Research Ethics Committee and therapists felt this may lead to diminution of the therapeutic relationship and/or inhibition so was not applied.

### **12. How well (actual fidelity)**

Most participants randomised to EMDR completed a full course of treatment as planned. Adherence was high, and the intervention was delivered in accordance with the adapted protocol following the approved modification to referral pathways.

**Appendix 3. Additional information regarding exploratory clinical outcome measures**

**Clinician-rated PTSD (CAPS-5)**

The Clinician-Administered PTSD Scale for DSM-5 (CAPS-5) was used to assess PTSD diagnostic status and symptom severity. The CAPS-5 is a structured clinical interview corresponding to DSM-5 PTSD criteria and is considered the gold-standard clinician-rated assessment of PTSD severity. Symptom frequency and intensity are rated on 5-point scales (0–4), yielding a total severity score ranging from 0 to 80. Higher scores indicate greater symptom severity (2).

In EMERALD, all CAPS-5 interviews were conducted by a single trained assessor (AB), who completed accredited CAPS-5 training and supervised practice prior to study commencement. Diagnostic decisions were made according to DSM-5 criteria using standard scoring algorithms. Interviews were conducted at 3 months (pre-randomisation baseline for the RCT cohort) and 12 months post-hospital discharge. The primary exploratory outcome was change in total CAPS-5 severity score between these timepoints.

**Self-reported PTSD symptoms (IES-R)**

The IES-R was used as the 2–3-month screening tool in EMERALD because it has been widely used in ICU survivorship research, was specified in the published trial protocol, and allowed comparability with previous post-ICU PTSD studies. A threshold of ≥22 was selected to prioritise sensitivity and reduce the risk of excluding survivors with clinically significant post-traumatic stress symptoms from the feasibility randomised trial. This threshold was used to identify a symptomatic group for further assessment and possible randomisation, not to establish a formal PTSD diagnosis (4).

The limitations of this approach are acknowledged. The IES-R was developed before DSM-5, is not equivalent to a DSM-5 PTSD diagnosis, and published cut-off scores vary across populations and clinical settings. In EMERALD, screening was not explicitly anchored to the ICU admission, meaning that some symptoms identified at screening may have reflected broader post-traumatic stress symptoms after critical illness rather than symptoms exclusively attributable to ICU exposure. Participants meeting the screening threshold therefore underwent subsequent CAPS-5 assessment before randomisation, enabling clinician-rated assessment of PTSD symptom severity and diagnostic status according to DSM-5 criteria.

In a definitive trial, we would retain a sensitive staged screening model but use clearer trauma-anchored screening instructions and evaluate DSM-5-aligned tools such as the PCL-5, alternative thresholds, and concordance between screening scores, CAPS-5 severity, and patient-prioritised symptom burden.

**Depression (PHQ-9)**

Depressive symptoms were assessed using the Patient Health Questionnaire-9 (PHQ-9), a 9-item self-report measure aligned with DSM criteria for major depressive disorder. Items are scored 0–3, yielding a total score between 0 and 27. Established cut-offs indicate mild (5), moderate (10), moderately severe (15), and severe (20) depression (5). The PHQ-9 has been widely used in medical populations and was administered at baseline, 3 months, and 12 months.

**Anxiety (GAD-7)**

The Generalised Anxiety Disorder-7 (GAD-7) is a 7-item self-report measure assessing core anxiety symptoms over the preceding two weeks. Items are scored 0–3, producing a total score between 0 and 21. Cut-offs of 5, 10, and 15 represent mild, moderate, and severe anxiety, respectively (6). The GAD-7 was administered at baseline, 3 months, and 12 months.

**Health-related quality of life (EQ-5D-5L)**

Health-related quality of life was measured using the EuroQol five-dimension, five-level instrument (EQ-5D-5L). The EQ-5D-5L assesses mobility, self-care, usual activities, pain/discomfort, and anxiety/depression across five response levels (7). Responses generate a health state profile convertible to a utility index using UK population tariffs (8). Participants also completed the EQ visual analogue scale (EQ-VAS; 0–100). The EQ-5D-5L was administered at baseline, 3 months, and 12 months to inform exploratory economic evaluation and to capture broader functional recovery.

**Adverse events**

Serious adverse events (SAEs) were defined as death, life-threatening events, hospitalisation or prolongation of hospitalisation, events resulting in persistent disability, or other medically significant events. Non-serious adverse events (AEs) included any untoward medical or psychological occurrence during trial participation, regardless of causality. AEs and SAEs were identified through participant self-report, therapist report, and review of medical records where applicable. No treatment-related serious adverse events were observed.

**Appendix 4: Theoretical Framework of Acceptability (TFA): Therapist perspectives on delivering EMDR**

| **TFA construct** | **Sub-themes (therapists)** | **Exemplar therapist quotes** |
| --- | --- | --- |
| **Affective attitude** | **Pride and purpose in innovation:** EMERALD seen as new, meaningful work in an under-served population. | “Really an exciting thing to be part of… new and innovative… so I was kind of all in.” (T1) • “I feel very proud to be part of this. It feels like this is a meaningful piece of work.” (T2) |
|  | **Therapeutic relationship as emotionally rewarding:** strong alliances with ICU survivors reinforcing positive affect. | “It’s been really delightful to work with this population.” (T2) • “It’s something I love doing… helps clients coming out of ITU.” (T3) |
|  | **Therapeutic structure and professional satisfaction:** time-limited, protocolised work experienced as containing and satisfying. | “It’s good… our usual caseloads are usually very complex trauma, long term.” (T3) • “There’s been lots of learning for me.” (T2) • “I really like the supervision, I’m learning so much.” (T1) |
|  | **Evolving engagement and negotiated familiarity:** initial apprehension easing into enjoyment and confidence. | “After the first couple of sessions, when I finally got to grips with it… I really enjoyed it.” (T4) • “Initially I wasn’t sure, but I feel excited about being part of the study.” (T5) |
|  | **Therapeutic fit and research optimism:** emotional investment linked to perceived clinical benefit and future impact. | “The clients that I’ve worked with have been very appreciative and they’ve really understood the impact the EMDR has had.” (T2) • “If it works… if it helps people or just informs further research… how good could this be?” (T3) • “Definitely happy. I can’t see another therapy being more effective than this one.” (T5) |
| **Burden** | **Emotional labour and clinical complexity of ICU survivors** – therapy-naïve, ambivalent patients requiring extra engagement work. | “So yeah, there was a lot more kind of digging around and reassuring them… some of them would be a bit more, like, apologetic…” (T4) • “It’s been challenging to work with people who are not used to a mental health setting.” (T2) |
|  | **Protocol fidelity vs therapeutic responsiveness** – anxiety about “doing it right” while flexing the protocol for complex presentations. | “I think there’s a bit of anxiety around ‘am I doing this as I should? Is it right? Am I doing it in the right way…’” (T3) • “That sort of thing might get in the way of continuing the usual protocol… but I think I’m pretty good at sticking to the protocol.” (T1) |
|  | **Procedural and administrative demands** – trial documentation and forms as a manageable but real learning curve. | “It was just getting used to where the information is on the forms and stuff… it’s kind of getting familiar with that.” (T5) • “The diary cards are helpful. The session templates we used… that was hugely helpful for me… I still follow it.” (T4) |
|  | **Relational and logistical burden** – external constraints (appointments, health) and the cognitive load of ICU material. | “They’ve got too many appointments in and around just getting better.” (T2) • “Luckily, we had supervision, otherwise I wouldn’t have known how to deal with that.” (T1) • “He’s trying to figure out which were real and what wasn’t real… it seems so real and he’s really wanting me to understand how real it was…” (T3) |
| **Ethicality** | **Personal and professional moral alignment** – EMERALD experienced as ethically congruent and socially valuable. | “Feeling really like this is really important to other people as well as it is to me. And I really like that.” (T4) • “I feel very proud to be part of this. It feels like this is a meaningful piece of work.” (T2) • “…such a valuable thing to be able to offer to clients in those situations.” (T5) |
|  | **Ethical tension around randomisation and the control arm** – distress at treatment allocation balanced against the need for evidence. | “When they get randomised as treatment as usual… that hurts my soul a little bit.” (T2) • “…if they’ve been told they may get it and then it’s like ‘oh, sorry’… yeah… I wonder how they feel.” (T3) • “You can’t go forward and develop good working practices… if you haven’t got evidence.” (T3) |
|  | **Informed consent and readiness** – concern that altruism and gratitude might mask ambivalence or limited understanding. | “Expectations… people have been maybe unsure of what it was they were coming into.” (T1) • “They were like, oh, yeah, of course we’ll do that to help you out, but didn’t maybe really understand… how much they would have to give of themselves.” (T1) • “I’m not confident yet that we fully understand this population well enough to say that we did everything we could at every stage.” (T2) |
|  | **Equity, access, and systemic gaps** – EMERALD seen as addressing a service gap in post-ICU psychological care. | “They might not get much support from the GP or might not be aware… this is the reason why I’m this, because I’ve had this admission.” (T4) • “They come out (of hospital) feeling like they should be grateful to be alive… and then it turns out it’s pretty rough.” (T1) |
|  | **Ethicality linked to observed benefit** – transforming outcomes framed as ethically compelling. | “It’s enough to help them with a sort of psychological strength, to carry on.” (T3) • “It’s released them from things that have been affecting their life for many years.” (T2) |
| **Intervention coherence** | **Conceptual fit and clinical justification** – EMDR seen as logically and technically suited to ICU trauma. | “It makes sense to me. Perfect sense. It’s target and short… a great fit.” (T3) • “What they’ve come with is that disturbance… absolutely, it’s the most effective quick way of managing that.” (T1) • “It can be pretty radical for people… pretty brief.” (T2) |
|  | **Understanding the wider trauma network** – EMDR revealing links between ICU trauma and earlier life experiences. | “It starts with things directly related (to ICU) and then it tracks back to those, very young, aged experiences.” (T5) • “What was really helpful… getting some more information about the experience, a typical experience of ITU.” (T2) |
|  | **Divergence and complexity in expectations** – moments of doubt when processing was unexpectedly “easy” or clients seemed “softer”. | “I suppose I was thinking they were a bit, a very softer client group.” (T4) • “Sometimes the ease of which the processing happened was kind of like… has that worked?” (T1) • “It’s been challenging to work with people who are not used to a mental health setting, but some of my misconceptions have been challenged.” (T2) |
|  | **Structure and flexibility** – valuing the EMDR frame while flexibly adapting within it. | “I like the model… it helps contain me as well… gives me a focus of what I’m doing.” (T3) • “I might use flash with him… but I’ll stick to the model.” (T4) • “You’re building the bridge with your therapy skills… and then able to join all the dots.” (T5) |
|  | **Therapeutic relationship as coherence scaffold** – alliance helping clients tolerate a “weird” intervention and therapists to deliver it confidently. | “The relationship really was key within the treatment.” (T4) • “Even when clients found the therapy ‘a bit wacky and weird’, they valued the opportunity to talk.” (T5) |
| **Opportunity costs** | **Integration into existing practice** – EMDR described as “slotting in” rather than displacing other work. | “It just very much slotted into my workload here. Little bit of like reading up in the evening prep… but I do that anyway.” (T4) • “In fact, it’s quite… easier because you don’t have to do all the extra bits… with clients that we’re seeing for different reasons… I love it.” (T3) |
|  | **Professional value and skill development** – EMERALD seen as an opportunity rather than a sacrifice. | “So, for my training it might be helpful to me as well to take part in it, which it… yeah really has been.” (T4) • “…because I did it as an extra… it was kind of joyous… just because it’s such a great therapy to do and effective.” (T1) |
|  | **Delivery constraints and shared pressures** – external constraints (work, hospital, therapist workload) occasionally limiting delivery. | “They literally couldn’t do it on a practical level because of work or hospital. They’ve got too many appointments in and around.” (T2) • “I’d only just returned to full-time duty and taking this on felt too much that time.” (T5) |
|  | **Engagement and relational trade-offs** – limits of therapeutic reach where clients ration their engagement. | “He’s like, I’ve done what you were set out to do… all the other stuff is old stuff that he doesn’t really want to go near.” (T1) • “They do like to chat. Maybe sometimes a cognitive analytical therapy or something like that would’ve been useful to more deeply understand his relationships.” (T1) |
|  | **Support and information reducing perceived cost** – supervision and ICU information seen as crucial to managing demand. | “When you send the [critical care] diaries over… copy of that would have been really helpful.” (T2) • “Luckily we had supervision otherwise I wouldn’t have known that.” (T1) |
| **Perceived effectiveness** | **Functional recovery and behaviour change** – observable improvements in travel, driving, independence, and reduced reassurance-seeking. | “They booked a holiday… talking about this being the first of many. Motivation had increased significantly.” (T4) • “More confident… dealing with issues, driving independently… not just dwelling on the past.” (T3) |
|  | **Broader psychological benefits and integration** – deeper processing, honesty with self, and reduced health service use. | “Hold on to those key emotions or sensations or thoughts… It’s like, wow, I haven’t thought about that for years.” (T5) • “It had a massive effect on him in terms of his honesty with himself.” (T1) • “It might stop them from seeking so much reassurance from other health providers and may actually reduce the rate of visits.” (T3) |
|  | **Conceptual coherence reinforcing perceived effectiveness** – ICU phenomenology seen as well-matched to EMDR mechanisms. | “Not everybody likes to do a lot of talking… this gives people the opportunity.” (T3) • “It starts with things directly related (to ICU) and then it tracks back to very young experiences.” (T5) • “I can’t see another therapy being more effective than this one.” (T5) |
|  | **Critical reflection and measurement limitations** – concerns about SUDS, reluctance to address non-ICU issues, and incongruence between reports and presentation. | “They were very much like, yeah, everything’s fine… but it’s not.” (T4) • “Yeah, ICU-related trauma was probably dealt with... just the other stuff... some reluctance to go back there.” (T1) • “His reporting of SUDS is not very effective… but objectively there are significant changes.” (T3) |
|  | **Professional conviction** – strong collective belief in EMDR’s appropriateness and impact for this population. | “I would want everybody to have the EMDR.” (T2) • “No doubts about that in any shape or form… just so valuable.” (T5) • “It really helps to unblock all of that... she felt... new lease of life and just being let go of so much baggage.” (T3) |
| **Self-efficacy** | **Early uncertainty and subsequent growth** – initial anxiety about fidelity and simplicity giving way to confidence. | “I was a little bit nervous, bit anxious. Was I doing it right…? There had to be more to it.” (T4) • “Getting together for more formal supervision… has been really helpful.” (T4) |
|  | **Protocol as containment** – structure and templates enhancing therapists’ sense of control and clarity. | “I like the model… it helps contain me… gives me a focus.” (T3) • “…the templates and diary cards were hugely helpful in scaffolding early sessions.” (T4) |
|  | **Relational efficacy** – confidence expressed through ability to use the relationship to work around avoidance and still make progress. | “He didn’t want to do any processing… but he wanted to see me.” (T4) • “Whether I’ve got that information or not… then the process of the therapy is going to take me there.” (T5) |
|  | **Confidence through supervision and peer reflection** – trial framed as a vehicle for professional development, not a threat to competence. | “It’s been nice to… just have that reflection with my peers.” (T1) • “I just eased into it, and I really enjoyed it… (with my supervisor) we can work with that and work through it.” (T4) |

**Appendix 5. Theoretical Framework of Acceptability (TFA): Participant perspectives on receiving EMDR**

| **TFA construct** | **Sub-themes (participants)** | **Exemplar participant quotes** |
| --- | --- | --- |
| **Affective attitude** | **Scepticism to openness** – unfamiliarity with EMDR initially met with doubt or uncertainty, often transforming into openness. | “I thought ‘what a load of nonsense’… then I looked it up and thought, oh, OK, this is not a newfangled thing.” (P2) • “I was sceptical… you don’t know the person… I did point out I’m on the autistic spectrum.” (P3) |
|  | **Relief, safety, and feeling understood** – relational safety was central; emotional containment enabled trust and disclosure. | “I’d become upset and she’d hold my hand, say everything’s OK.” (P7) • “She could see when I was really hammered… she was excellent.” (P20) |
|  | **Emotional ambivalence and exposure discomfort** – therapy often helpful but emotionally taxing, and at times distressing. | “I only found it difficult because I was having to remember certain things…” (P19) • “Sometimes… I found that repetitive and distressing.” (P1) |
|  | **Enjoyment, routine, and connection** – EMDR became a valued weekly anchor, linked to relief, structure, and meaningful contact. | “I always used to leave there feeling like I just offloaded a lot of stuff.” (P7) • “Very pleasant place… I think it got me out.” (P2) |
|  | **Altruism and purpose** – many framed participation as giving back to ICU services and future survivors. | “The main reason… was a giving back sort of thing to ICU.” (P16) • “Anything I can do to help other people I am willing to do.” (P8) |
| **Burden** | **Practical burden low** – attending sessions (online or face-to-face) generally straightforward. | “We’re on Zoom… it was fine.” (P11) • “I looked forward to the sessions… face-to-face helped.” (P8) |
|  | **Cognitive effort and fatigue** – emotional intensity, anticipatory dread, and post-session exhaustion. | “It’s like your brain is frying itself in there.” (P20) • “I knew it was coming… I didn’t really want to do it today.” (P16) |
|  | **Challenges with specific EMDR components** – discomfort or difficulty with eye movements, imagery, or tapping. | “I didn’t like the eye movement.” (P8) • “I’m not quite so good at imagining random things.” (P3) |
|  | **Therapy setup and environmental factors** – minor technical or contextual issues influenced burden. | “We had to work out where I was going to sit with the camera…” (P3) • “Hospital appointments… but no other issues.” (P19) |
| **Ethicality** | **Personal relevance and legitimacy** – EMDR widely viewed as appropriate and meaningful for post-ICU recovery. | “I know why this has happened… this should happen for every patient disturbed by ICU.” (P16) |
|  | **Alignment with recovery values and responsibility** – EMDR linked to resilience, agency, and doing what is needed to recover. | “She said we’ll deal with that as well… I was very glad.” (P3) • “What kind of burden would that have meant if I hadn’t had it?” (P16) |
|  | **Value of relational qualities** – ethical acceptability tethered to trust, compassion, and safety within the therapeutic relationship. | “I must have felt safe… we went into things I haven’t talked about in years.” (P3) • “She would hold my hand… I felt completely at ease.” (P7) |
|  | **Absence of ethical conflict** – no objections raised on cultural, religious, or moral grounds. | “No ethical issues at all.” (P11) • “I wasn’t afraid of it at all.” (P20) |
| **Intervention coherence** | **Initial confusion and unfamiliarity** – EMDR initially perceived as odd, unclear, or gimmicky. | “I didn’t understand it at all… just thought I put my eyes left, right…” (P11) • “At the beginning… it was a little bit jokey.” (P7) |
|  | **Progressive understanding through experience** – coherence developing gradually through doing rather than explaining. | “Once you get past that… it’s great.” (P7) • “As we got more into it, I understood it a little bit better.” (P11) |
|  | **Personalised adaptation and meaning-making** – modifying imagery and technique improved coherence. | “I put mine in a clinical waste bag and buried it…” (P3) • “I’d think about this safe place… eventually I’d fall asleep.” (P19) |
|  | **Therapist guidance and process trust** – coherence supported by pacing, reassurance, and relational confidence. | “She explained it to me and made me feel like it was OK.” (P7) • “She had to control the sessions going too deep… and it helped.” (P16) |
| **Opportunity cost** | **Emotional avoidance and psychological cost** – some avoided the “worst stuff” to protect themselves from distress. | “I avoided the worst stuff… I couldn’t go there.” (P8) • “Repetitive and distressing.” (P1) |
|  | **Physical and practical inconvenience** – fatigue, discomfort, technical distractions particularly in remote sessions. | “I had a serious itch on my leg… I have to stop this now.” (P3) • “Sometimes it really drained you… but it did get me places.” (P20) |
|  | **Value outweighing cost** – participants emphasised benefits far exceeding any inconvenience or distress. | “The NHS would be mental not to pick this up.” (P16) • “I don’t know where I’d have been without it.” (P1) |
| **Perceived effectiveness** | **Symptom relief and functional gains** – reductions in flashbacks, improved sleep, returning to work. | “I don’t get flashbacks.” (P11) • “My sleep pattern got better and better.” (P16) • “There’s some distance between me and what happened.” (P1) |
|  | **Emotional processing and integration** – EMDR enabled containment and reorganisation of distressing experiences. | “It’s got a lid on and it’s sealed.” (P16) • “I’ve got a little blue box… where I put all the bad things.” (P20) |
|  | **Therapeutic alliance as mechanism of change** – therapist expertise and empathy seen as central to effectiveness. | “A consummate professional… she gets the balance just right.” (P1) • “Someone was listening to me… someone who understood.” (P19) |
|  | **Cognitive shift and letting go** – improvement linked to releasing analytical control and tolerating uncertainty. | “Shut my mind off and be in the moment.” (P3) • “Those things are still with me… but they’ve become part of me.” (P1) |
|  | **Limitations and ongoing struggles** – partial improvement where trauma or comorbidity was more complex. | “I still don’t know exactly what happened… I just can’t open up.” (P8) • “I was probably one of those complicated people…” (P7) |
| **Self-efficacy** | **Clarity and readiness to engage** – some participants felt prepared and confident from the outset. | “I knew what was expected of me… I participated well.” (P11) |
|  | **Letting go and trusting the process** – confidence increased when relinquishing control and engaging experientially. | “Shut my mind off and… be in the moment.” (P3) • “I’d start to get mindful as soon as I left… and leave it behind me.” (P1) |
|  | **Therapeutic support enabling confidence** – relational containment scaffolded participants’ ability to engage. | “She took that on board… gave me other outlets.” (P3) • “Someone was listening… someone who understood.” (P19) |
|  | **Barriers to full participation** – mistrust, guardedness, or fear of re-traumatisation limiting consistency or depth. | “I can be stubborn… I didn’t wanna go to those dark places.” (P8) • “I was trying to stop my brain from remembering.” (P19) |
|  | **Adaptation, ownership, and control** – active modification of techniques to enhance safety and agency. | “I put mine in a clinical waste bag and buried them…” (P3) • “I managed to switch the brain off so that I could block it all out.” (P19) • “I thought she was in the ward next to me… I was able to adjust and go ‘this may not be real’.” (P7) |

**Appendix 6. Exploratory baseline-adjusted analysis of clinician-rated traumatic stress severity**

Because CAPS-5 total scores were higher in the EMDR group than in the treatment-as-usual group at RCT baseline, an exploratory baseline-adjusted analysis was undertaken. An analysis of covariance model was fitted with 12-month CAPS-5 total score as the dependent variable, treatment group as the independent variable, and baseline CAPS-5 total score as a covariate. Model residuals were inspected visually and did not suggest substantial departures from normality or homoscedasticity.

After adjustment for baseline CAPS-5 total score, the estimated 12-month CAPS-5 total score was lower in the EMDR group than in the treatment-as-usual group, with an adjusted mean difference of −11·08 points (95% CI −18·97 to −3·19). Baseline CAPS-5 total score was also associated with 12-month CAPS-5 total score. This analysis was exploratory and should not be interpreted as a definitive test of treatment effectiveness because the trial was not powered for clinical effectiveness and the sample size was small.

| **Model term** | **Estimate** | **95% CI** | **p value** |
| --- | --- | --- | --- |
| Treatment group: EMDR versus treatment as usual | −11·08 | −18·97 to −3·19 | 0·007 |
| Baseline CAPS-5 total score | 0·48 | 0·03 to 0·93 | 0·037 |

***Table note:*** *The dependent variable was 12-month CAPS-5 total score. The treatment-group estimate represents the adjusted mean difference for EMDR minus treatment as usual. Negative values favour lower clinician-rated PTSD symptom severity at 12 months. CAPS-5=Clinician-Administered PTSD Scale for DSM-5; EMDR=eye movement desensitisation and reprocessing; CI=confidence interval.*

**Appendix 7: Joint display of feasibility, quantitative outcomes, and qualitative acceptability findings structured by the Theoretical Framework of Acceptability (TFA)**

| **TFA Construct** | **Quantitative Findings (Feasibility + Clinical Outcomes)** | **Qualitative Findings (Participants + Therapists)** | **Integration (Convergence / Dissonance)** |
| --- | --- | --- | --- |
| **Affective attitude** | Strong feasibility signals: 77% (40/52) of eligible survivors consented to randomisation; 98% (39/40) retention at 12 months; 90% (18/20) of EMDR-allocated participants initiated therapy; 75% (15/20) completed the full course. | **Participants**: Initial scepticism often gave way to trust, safety, and emotional relief; sessions described as valued routine and source of connection. **Therapists**: Feelings of pride, purpose, and emotional fulfilment; rewarding relationships; initial uncertainty diminishing with experience. | **Convergent**: High engagement metrics align with predominantly positive emotional evaluations. Minor early ambivalence in both groups did not hinder uptake. Quantitative retention mirrors qualitative accounts of trust, safety, and emotional reward. |
| **Burden** | Low practical burden: most participants attended regularly; attrition unrelated to therapy acceptability. One withdrawal due to time constraints; otherwise high adherence. | **Participants**: EMDR generally easy to attend and integrate into routines; some emotional fatigue and cognitive effort; occasional difficulties with visualisation or exposure tasks. **Therapists**: Emotional labour in a therapy-naïve population; procedural learning curves; manageable protocol demands with supervision. | **Convergent**: Both datasets show EMDR as low burden with tolerable emotional effort. Qualitative nuance (emotional fatigue, administrative load) did not manifest as dropout quantitatively. Overall burden acceptable and non-disruptive. |
| **Ethicality** | No ethical objections identified. High consent rate and absence of values-related withdrawal suggest ethical acceptability. | **Participants**: EMDR seen as appropriate, legitimate, and aligned with personal recovery values; altruism prominent. No cultural/religious objections. **Therapists**: Strong moral alignment; valued addressing a neglected clinical need; some discomfort about control group allocation but acceptance of evidence-generation rationale. | **Convergent**: Ethical congruence across modalities. Participants and therapists saw EMDR as appropriate, purposeful, and morally aligned. Minor therapist concerns about randomisation did not impair trial delivery. |
| **Intervention coherence** | Indirect quantitative indicators: high initiation 18/20 (90%) and high programme completion 15/20 (75%) suggest sufficient understanding for engagement; no delays or failures related to misunderstanding of EMDR. | **Participants**: Early confusion common, resolving through experience; personalised meaning-making; therapist explanation key. **Therapists**: Strong conceptual understanding; perceived clinical fit; coherence occasionally challenged by unexpected ICU-related complexity but reinforced via supervision and therapeutic structure. | **Convergent**: Participants initially uncertain but attained workable understanding; therapists confident about conceptual fit. Quantitative fidelity and session completion confirm that limited initial coherence did not impair delivery. |
| **Opportunity costs** | Minimal: only one time-constraint withdrawal in EMDR arm; participants did not report giving up valued activities; EQ-5D data showed no functional loss. | **Participants**: Minor logistical or emotional costs (fatigue, scheduling) but outweighed by perceived benefit; occasional avoidance of highly distressing material. **Therapists**: EMDR integrated smoothly into workload; low administrative cost; some constraints from participant availability and ICU-related complexity. | **Convergent**: Opportunity cost low across datasets. Small qualitative frictions did not translate into meaningful quantitative burden. EMDR viewed as efficient and worthwhile. |
| **Perceived effectiveness** | **Clinical outcomes**: EMDR associated with larger reductions in CAPS-5 total score and diagnostic resolution vs TAU; effect size meaningful but imprecise due to feasibility sample. PROMs showed modest improvements but no clear between-group differences. | **Participants**: Strong perceived symptom relief (sleep, flashbacks, emotional stability), functional gains, reduced health anxiety. **Therapists**: Observed behavioural recovery, deeper processing, and improved daily functioning; some complexity and residual difficulties noted. | **Mostly convergent**: Qualitative narratives strongly endorse meaningful clinical benefit, consistent with CAPS-5 changes. PROMs less sensitive to change, suggesting complementary measurement. Both sources highlight functional and emotional gains. |
| **Self-efficacy** | Engagement fidelity high: 18/20 (90%) initiated EMDR, 15/20 (75%) completed; only one participant unable to engage due to access issues. | **Participants**: Confidence varied; readiness increased over time; some needed support with exposure or “letting go”; adaptations enhanced ownership. **Therapists**: Confidence increased with supervision and familiarity; structured EMDR model supported delivery. | **Convergent**: Both datasets show steadily increasing confidence. Therapist support and structured delivery enhanced participant self-efficacy. Quantitative completion data align with qualitative progression from uncertainty to capability. |

**References**

1. Hoffmann TC, Glasziou PP, Boutron I, et al. Better reporting of interventions: template for intervention description and replication (TIDieR) checklist and guide. *BMJ* 2014; 348: g1687.
2. Weathers FW, Bovin MJ, Lee DJ, et al. The Clinician-Administered PTSD Scale for DSM-5 (CAPS-5): development and initial psychometric evaluation in military veterans. *Psychol Assess* 2018; 30: 383–95.
3. Weiss DS. The Impact of Event Scale–Revised. In: Wilson JP, Tang CS, eds. Cross-cultural assessment of psychological trauma and PTSD. Boston, MA: Springer; 2007: 219–38.
4. Rash CJ, Coffey SF, Baschnagel JS, Drobes DJ, Saladin ME. Psychometric properties of the IES-R in traumatized substance dependent individuals with and without PTSD. *Addict Behav* 2008; 33: 1039–47.
5. Kroenke K, Spitzer RL, Williams JB. The PHQ-9: validity of a brief depression severity measure. *J Gen Intern Med* 2001; 16: 606–13.
6. Plummer F, Manea L, Trepel D, McMillan D. Screening for anxiety disorders with the GAD-7 and GAD-2: a systematic review and diagnostic meta-analysis. *Gen Hosp Psychiatry* 2016; 39: 24–31.
7. Devlin N, Pickard S, Busschbach J. The development of the EQ-5D-5L and its value sets. In: Devlin NJ, Pickard AS, Busschbach JJV, eds. Value sets for EQ-5D-5L: a compendium, comparative review and user guide. Cham: Springer; 2022: 1–12.
8. Devlin NJ, Shah KK, Feng Y, Mulhern B, van Hout B. Valuing health-related quality of life: an EQ-5D-5L value set for England. *Health Econ* 2018; 27: 7–22.
